# Intelligent health promotion system-based construction and implementation of exercise intervention for older adults with frailty and prefrailty

**DOI:** 10.3389/fragi.2026.1780093

**Published:** 2026-08-13

**Authors:** Mengya Liu, Li Zhang, Qin Huang, Xiaoli Zhu, Yu Wang, Lin Zhu

**Affiliations:** 1 Department of Gastroenterology,Xuyi,s People Hospital, Xuyi, China; 2 School of Nursing, Bengbu Medical University, Anhui, China; 3 Xuyi,s People Hospital, Xuyi, China

**Keywords:** aging adults, comprehensive framework for implementation research, exercise intervention, frailty, old adult

## Abstract

**Background:**

Frailty in the elderly population is increasing, and the development of high-quality clinical practice guidelines or consensuses can guide medical staff to better provide health services for frail elderly individuals. In recent years, scholars at home and abroad have actively explored exercise intervention management in elderly individuals with frailty or prefrailty. How to rapidly generalize interventions with “known and definite effects” for the community-dwelling frail elderly population is the core issue of the research on frailty. There is a lack of study regarding whether digital interventions can improve exercise adherence for older adults with frailty and prefrailty.

**Objective:**

In accordance with the Comprehensive Framework for Implementation Research (CFIR), this study implemented scientific strategies for exercise interventions for elderly people with community frailty and prefrailty. Empirical research was conducted via the cloud platform of the home‒community integration system to provide a theoretical basis for improving the physical, psychological and social frailty of frail elderly people and reducing the incidence of adverse outcomes caused by frailty in the community.

**Methods:**

Qualitative and quantitative research methods were fully integrated. Semistructured interviews were conducted with 64 older adults who designated family doctors and 44 community workers in the community to identify the regular exercise adherence of frail or prefrail older adults as well as the promoting and hindering factors of community workers in the implementation of scientific exercise interventions. Following the guidance of the Comprehensive Framework for Implementation Research (CFIR), scientific exercise intervention strategies for frail elderly people in the community were implemented. The control group received routine nursing, and the intervention group received a scientific nursing intervention. After 3 months of intervention, the effects of the strategy on implementation outcomes (feasibility, acceptability, fidelity, etc.), intervention outcomes (physical function, emotional state, social support, self-efficacy), process outcomes (skills or cognition), and resource utilization in older adults with frailty or prefrailty were evaluated.

**Results:**

In the qualitative interviews conducted with CFIR as the theoretical guiding framework, a total of 22 influencing factors, 12 promoting factors and 10 hindering factors in 4 areas were identified to construct scientific strategies for implementation。The implementation results revealed that the feasibility (P = 0.003), acceptability (P = 0.002) and fidelity (P = 0.001) of the intervention group were significantly better than those of the control group. The intervention results revealed that, compared with those in the control group, physical fitness (gait: P = 0.016; balance: P < 0.001; grip strength: P < 0.001; body fat percentage: P < 0.001), depression (P < 0.001), social support (P < 0.001), and self-efficacy (P < 0.001) significantly improved**.**

**Conclusion:**

Guided by the implementation of the scientific theoretical framework, this study used a whole-process, systematic, diversified and scientific online and offline joint exercise implementation strategy, proving that the strategy can be used to create a safe, effective and sustainable exercise intervention for frail or prefrail elderly individuals in the community that effectively improves their physical fitness, depression, social support and self-efficacy, delays the progression of frailty, and reduces the incidence of adverse outcomes such as disability, falls, hospitalizations and even death.

## Background

Elderly people constitute a special population, and the manifestations of aging are highly heterogeneous among older individuals (Wu et al., 2022). Age is no longer an accurate indicator of aging or declining physical function in older adults; instead, frailty is the main factor that leads to individual differences in aging (Zhu, 2021). Approximately 10% of community-dwelling adults aged 60 years and older in China are in a state of frailty, and approximately 25% of those over 85 years of age are in a state of frailty (Ma et al., 2018; Liang et al., 2019). Frailty is an age-related syndrome characterized by decreased physiological reserve and impaired anti-stress capacity resulting from the accumulation of age-related multisystem decline (Geriatrics Branch of Chinese Medical Association and Editorial Board of Chinese Journal of Geriatrics, 2022; Feng et al., 2023). Disease progression can lead to a range of adverse events, such as decreased function, falls, disability, hospitalization, and increased risk of death, as well as the depletion of medical resources and the increased burden of home care needs (Perez-Zepeda et al., 2020; Liu et al., 2021). How to take effective measures to prevent frailty has become a hot topic in geriatric research. Achieving healthy aging requires the efforts of society as a whole to move the threshold forward and slow the progress of frailty through active interventions at the primary healthcare level. Early exercise intervention in older adults has been shown to be the most critical measure for delaying or reversing frailty in older adults (Du, 2024). Exercise can improve physical function, emotional state, and social support in frail older adults.

Frailty in the elderly population is increasing, and the development of high-quality clinical practice guidelines or consensuses can guide medical staff to better provide health services for frail elderly individuals. In recent years, scholars at home and abroad have actively explored exercise intervention management in elderly individuals with frailty or prefrailty. In 1975, the American College of Sports Medicine published the first edition of the ACSM Guidelines for Exercise Testing and Exercise Prescription, which advocated that “exercise is good medicine” and provided exercise guidance for frail and prefrail older adults (Haskell et al., 2007). In 2017, geriatric experts in the Asia–Pacific region issued clinical practice guidelines for the management of frailty in the Asia–Pacific region (Dent et al., 2017), which highly recommended sports with resistance training components. In 2019, the International Society for the Study of Frailty and Sarcopenia published guidelines for the International Conference on Frailty and Sarcopenia (Dent et al., 2019), which state that a multicomponent physical exercise program that includes resistance training is strongly recommended as a first-line treatment for the management of frailty and health practitioners are encouraged to recommend progressive, resistance training programs for frail older adults. In 2022, the Chinese Geriatric Association issued a Chinese expert consensus on the prevention of frailty in elderly individuals, recommending that multiple exercise programs be safely and effectively integrated into the lives of frail elderly individuals. In addition, to promote the localization of relevant guidelines in China, the Hefei Institute of Physical Sciences of the Chinese Academy of Sciences, in cooperation with numerous hospitals and colleges and based on the American College of Sports Medicine and the Asia–Pacific guidelines, issued the Technical Specifications for Sports Promotion Health Services–Local Standards of Anhui Province (Sun et al., 2021), which standardized the definition of personalized exercise prescription and other concepts, considered local community resources and the characteristics of the elderly population, and proposed corresponding scientific fitness exercise guidance programs for elderly people with different disease characteristics. New sports health promotion technology uses intelligent decision-making technology to generate safe and reliable personalized exercise prescriptions and deploy them in the smart health huts of community health service centers. This technology has achieved remarkable results, confirming the scientific effectiveness of the standard for localization.

How to rapidly generalize interventions with “known and definite effects” for the community-dwelling frail elderly population is the core issue of the research on frailty. To address this issue, the concept of implementation science has been proposed. Implementation science refers to methodological research that promotes the adoption of evidence-based practices, interventions, and policies and their integration into routine healthcare and public health settings to improve the impact on human health (Mandell, 2025; Zhang et al., 2024). As the name suggests, implementation science is an evidence-driven scientific approach or strategy that explores the enablers and impediments to the implementation of an intervention, reinforces and consolidates the enablers and uses the impediments it identifies to develop and adopt appropriate implementation strategies. In this study, the Consolidated Framework for Implementation Research (CFIR), the most commonly used implementation science framework, was used to conduct implementation studies of guidelines, which evaluated potential enablers and impediments from the implementation plan, external factors, internal factors, individual factors, and the implementation process. Exercise interventions have been repeatedly shown to delay frailty and prevent frailty-related adverse outcomes in older adults. Therefore, on the basis of the four dimensions of the CFIR (implementation intervention, external factors, internal factors and individual factors), this study used qualitative interviews to analyze the corresponding promoting and hindering factors in the implementation of scientific exercise programs. The goal was to construct a scientific personalized exercise intervention strategy that could be quickly implemented *via* a community health platform to improve the acceptance and implementation rate by community workers and improve the physical, psychological and social health status of frail and prefrail elderly individuals in the community, which is highly important for improving their quality of life and promoting healthy aging.

## Research methods

### Research framework

**FIGURE 1:**
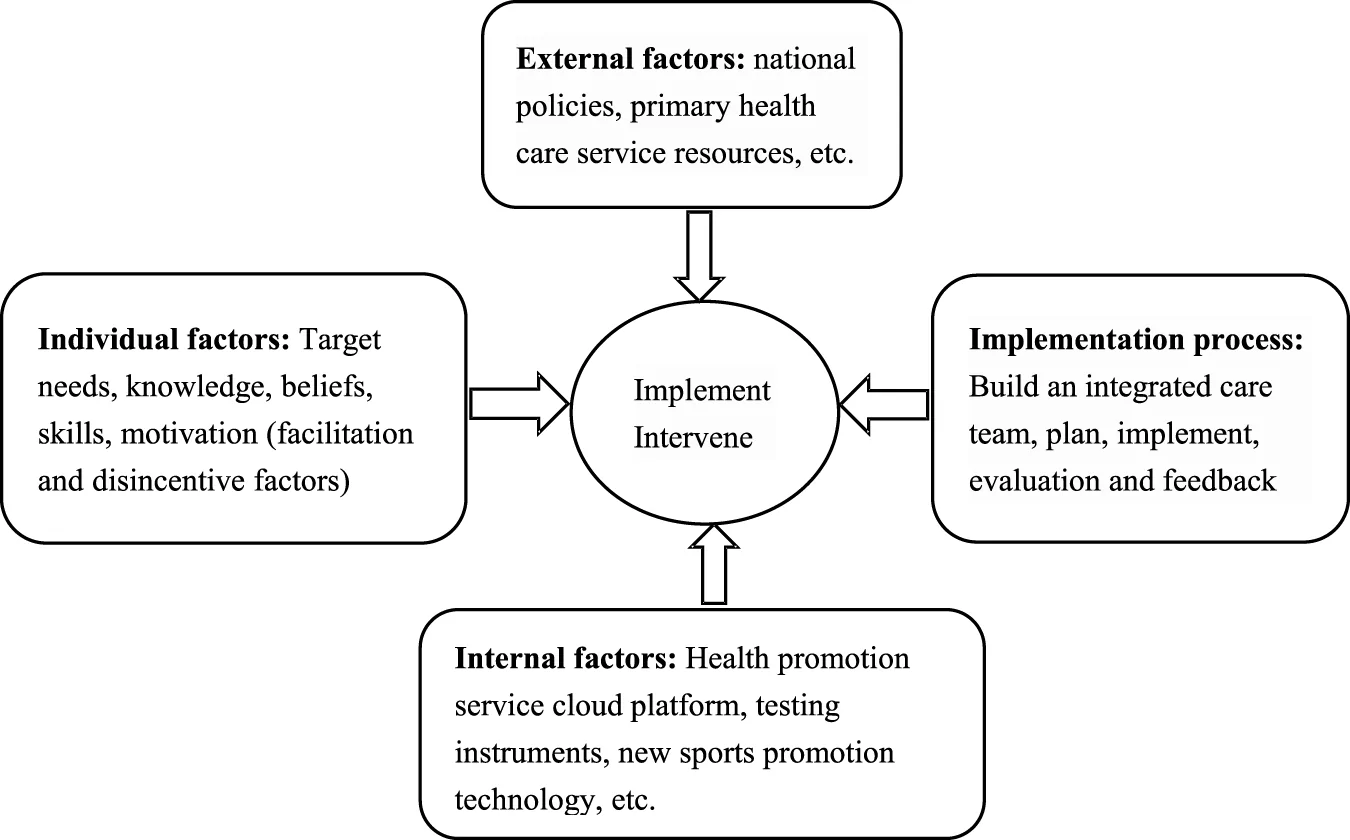
Framework for the implementation of scientific research.

The purpose of conducting scientific research is to explore the barriers to and facilitators of the normalization of evidence-based practices and then to adopt corresponding implementation strategies. The Comprehensive Framework for Implementation Research (CFIR) is the most widely used framework of determinants, including implementation plans, external factors, internal factors, individual factors, and implementation processes (Liu Y. et al., 2023; Zhang Q. et al., 2021; Qin et al., 2023; Yu et al., 2024). On the basis of an analysis of exercise interventions for frail or prefrail elderly individuals in the literature, this study adopts the theoretical framework of the implementation of scientific integration and integrates the five core elements mentioned above to form the framework of this study (Figure 1).

### Qualitative research

#### Data collection

First, the CFIR structure related to the implementation of frailty measures was identified, a semistructured interview outline was developed according to the CFIR interview guidelines, and in-depth interviews were conducted with frail or prefrail elderly individuals. The interview outline was based on the five domains of the CFIR theoretical framework and encouraged frail or prefrail older adults to identify the facilitators and impediments that affect regular exercise. Before the formal interviews, 10 interviewees were preinterviewed, and the interview outline was revised on the basis of the results. The interviews were conducted face-to-face, and the interviewer flexibly adjusted the order and method of the questions before the interview according to the interview outline and the interviewee’s situation. During the interviews, the interviewees were asked to listen carefully, and their body language was carefully observed. Before the interview, the purpose of the study was explained, and consent was obtained. Each interviewee was then interviewed for 20–40 min, and the entire process was recorded synchronously.

#### Data sorting and analysis

The data were sorted and analyzed *via* phenomenological research methods. Twenty-four hours after the interview, the recording materials were repeatedly listened to and transcribed word for word. The transcribed texts were read carefully, meaningful views were identified, extracted and summarized, similar concepts were searched, and themes were extracted. The analysis was conducted using 39 components of 5 CFIR fields as the coding templates, and the themes were projected into the CFIR framework fields and structures for category summaries. In cases of disagreement, the researcher returned to the interviewee for confirmation.

### Qualitative interview findings

#### General information on the interviewees

The respondents to the qualitative interviews in this study included elderly individuals and community workers in the Hubin and Dongfeng communities. There were 11 community-dwelling elderly people, including 5 women and 6 men. There were 7 people over 60 years old and 4 people over 70 years old. General information on the elderly people in the community is shown in Table 1. There were 9 community workers in total, including 3 nurses, 3 doctors, 2 volunteers and 1 rehabilitation physiotherapist. The general information of the community workers is shown in Table 2.

**TABLE 1:** General data of the elderly in the community (n = 11).

Number	Gender	Age	Residence	Marriage	Economy	Education	Chronic disease	Exercise habits
N1	Female	62	Rural	Married	1000	Junior	1	Yes
N2	Female	67	Rural	Married	400	Primary	2	No
N3	Male	77	Rural	Divorce	400	Primary	3	Yes
N4	Female	64	Rural	Married	3000	High	1	Yes
N5	Male	68	City	Married	4000	High	0	Yes
N6	Female	71	City	Married	2000	Junior	1	Yes
N7	Male	65	Rural	Married	1000	Junior	2	Yes
N8	Male	61	City	Married	4500	College	1	Yes
N9	Male	68	Rural	Married	2000	Junior	1	Yes
N10	Female	74	City	Widowed	400	Primary	3	No
N11	Male	70	Rural	Married	1000	Primary	1	Yes

**TABLE 2:** General information of community workers (n = 9).

Number	Gender	Age	Education	Job title	Posts	Years of service/Year
C1	Male	47	Graduate student	Deputy chief physician	Doctor	21
C2	Female	43	Graduate student	Deputy chief physician	Doctor	19
C3	Male	41	Graduate student	Deputy chief physician	Doctor	15
C4	Female	50	Graduate student	Deputy chief nurse	Nurse	27
C5	Female	58	Undergraduate	Nurse in charge	Nurse	32
C6	Female	48	Undergraduate	Nurse in charge	Nurse	22
C7	Female	45	Undergraduate	Assistant	Volunteer	19
C8	Female	31	Undergraduate	Assistant	Volunteer	17
C9	Male	36	Graduate student	Intermediate	Physiotherapist	12

#### Promoting and hindering factors of qualitative interviews

In the exercise intervention project implemented with CFIR as the theoretical guidance framework, through the qualitative interviews with the elderly people and community workers, 22 influencing factors for implementation in 4 fields were identified by consensus. Among these factors, 12 were promoting factors, and 10 were hindering factors, as shown in Table 3.

**TABLE 3:** Promoting and hindering factors of exercise intervention implemented under the theoretical guidance framework of CFIR.

CFIR framework	Promoting factors	Hindering factors
Intervention regimen	①Evidence-based guidelines: In the interviews, it was found that respondents expressed support for the intervention program from domestic and foreign exercise guidelines. C9: “The American Academy of sports medicine has long proposed exercise prescribing guidelines, and their effectiveness and safety have been validated and should be tried in our community.” C2: “This intervention plan is derived from the consensus or guidelines formulated by relevant experts at home and abroad, and is highly scientific, and now any intervention plan needs to be evidence-based.” ②Outstanding advantages: Most respondents believe that exercise interventions for the frail elderly can alleviate the pressure of community members. C8: “Exercise program: Regular exercise prescription training mode, through joint supervision by the family and the community, through the assessment of motor function through an electronic monitoring system, relieves the pressure in our community”.	①Adaptability and adaptability need to be improved: Respondents believe that intervention regimen and environments need to be adapted. C4: “We have tried these solutions before, but there will be many setbacks in the middle, and it may be necessary to optimize and adjust the solutions many times later to adapt them to the local environment.” C3: “In the process of implementation in our community, if the implementation effect is not good, the preliminary work may consume a lot of manpower and material resources.” ②Complexity of multi-team implementation: Respondents felt that the implementation of a scientific exercise intervention program was complex, requiring a multidisciplinary team to conduct a comprehensive assessment and personalized improvement of exercise prescriptions in real time. C1: “Doctors, nurses, *etc.*, will increase the work pressure in the follow-up implementation plan, and the follow-up also needs to be supported by project funds, otherwise it may increase the economic burden.”
External factors	①There is relevant policy support: “National fitness plan 2021–2015″, *etc.*,; the interview found that the country is currently vigorously promoting sports and exercise, and relevant policies have been introduced to support the promotion of sports. N5: “The state vigorously promotes sports for all, provides fitness venues and facilities, encourages us to exercise more, which is good for our health.” ②Peer pressure: Respondents said that peer pressure would give them a certain amount of motivation, which would facilitate the implementation of the project. C2: “At present, there are many relevant exercise guidelines and expert consensus for the elderly, which shows that exercise has been taken seriously, and now community hospitals in many large cities have developed and implemented scientific exercise projects and started to conduct pilot intervention trials, and we must also keep up with current events and start trying.”	①Lack of individualized exercise methods for the elderly: The physical activity guidelines for the Chinese population only propose that physical activity recommendations for adults are applicable to the elderly. N2: “Now that I’m older, how can I be like a young man, I’m very tired when I move now.” N10: “When I’m older, I do not know what exercise is right for me, and I get tired when I exercise, and I do not know how to exercise.” ②Lack of mass media awareness-raising: Most of the elderly have less information about sports and health. N2: “I’m not proficient in using my phone, and every time I watch a show on TV, or listen to someone say that I do not understand these things.”
Internal factors	①Peer effect: Respondents said that the peer effect gives them some motivation. N8: “My friend goes to the community every day to exercise, rain or shine, so he is in very good health, and we can’t be left behind.” ②Athletic atmosphere: Respondents said that an effective athletic atmosphere promotes perseverance and efficiency. N4: “We dance together every 2 days, and sometimes we make our own choreography and go to competitions.” ③Support and encouragement from family members: The majority of respondents believe that support and encouragement from their children will promote the continuation of sports. N9: “My daughter really wants me to exercise more every day, and often tells me to go out for a walk and square dance or something.” ④Community sports venues and facilities: There are many sports equipment in the Dongfeng community to help respondents exercise. N11: “We have built some sports equipment in our community, which is very convenient.” N9: “In the past, the neighborhood committee would issue elastic bands and teach us how to use them, so it’s good for me to pull them every day.” ⑤Support and encouragement from the community and school: Respondents felt that support and encouragement helped with the implementation of the project. C7: “Our community leaders and school teachers are very supportive of us in doing this project, and we can do it with confidence and seriousness.”	①Insufficient available resources: Aging equipment or insufficient human resources in some communities hinder the implementation of the campaign. N6: “Some of the equipment in our community is broken, and we dare not use it.” N7; “when there are so many people at night, we have nothing to do, so we can only move around by ourselves.” C1: “We have so few people in the team that we have a wide range of disciplines for this project, and we are certainly not enough to do it ourselves.” ②Lack of IT infrastructure for teaching: Interviews revealed that the promotion of smart health huts in the community is insufficient. N2: “The community hospital has that health hut, which is actually pretty good, but we won't use it.” C5: “The scope of information received by community nursing staff is somewhat limited and slow, except for nursing graduate students to check the progress of relevant research, the rest of the staff receive information almost exclusively from doctors in the department, and the available information is updated slowly.” ③Limited accessible motor knowledge: Older adults are limited and slow to receive relevant information. N1: “I live a little far away, and I do not usually hear of lectures in the community.” N2: “I have not been to the community lecture for a long time, and some knowledge is not often pushed on WeChat, and the update is very slow.”
Individual factors	①Self-efficacy: During the interview, it was found that the more confident the respondents were in their abilities, the easier it was to stick to exercise. N5: “I usually like to box, I go every day, rain or shine, I think I can last until I am 100 years old.” ②Exercise needs: Respondents need exercise for some reason. N3: “I have three highs, and the doctor said that I need to exercise more, exercise can maintain normal blood pressure, and I can lose weight, so I need to exercise more.” ③Individual maturity: Respondents said that as they were exposed to the intervention program, they became more proficient in understanding the intervention program and health education. C6: “At the beginning, we only collected relevant knowledge and information, but with the increase of relevant research and the extension of the pilot time, we were able to make a complete elaboration on the specific implementation details of the intervention plan and find out the deep-seated problems.”	①Lack of knowledge and skills in regular exercise: After the interview, it was found that most of the respondents thought that exercise was walking, walking, *etc.*, and did not understand resistance movement. N3: “The doctor told me to exercise more, to run every time, and I did not take the initiative to learn about this.” N10: “We know that sports are good, but no one teaches us, and the videos we usually watch do not feel practical, so it would be nice if there were professionals who taught us.” ②Lack of attitude and motivation: Lack of the right attitude and motivation can hinder the implementation of the exercise. N6: “Sometimes the weather is bad and I do not want to move.” N10: “I do not like sports, I like to watch TV, I do not have the motivation to exercise, I do not want to move.” ③Lack of individual identification with the team: Respondents indicated that a rapport between team members would lead to an individual’s willingness to go the extra mile for the team and facilitate the implementation of intervention projects. C9: “Everyone’s task is different, but the goal is the same, and each of us will only promote the smooth implementation of the intervention when the ultimate goal makes the same contribution or receives the same treatment, but at present, everyone’s task responsibilities and tasks are different.”

## Construction of a motor intervention implementation strategy

Internal and external environmental factors, individual factors, and the implementation of the intervention process can all affect the implementation of scientific sports programs. The semistructured interviews clarified the compliance of the frail and prefrail older adults in the community with regular exercise and the promoting and hindering factors of community workers in the implementation of scientific exercise interventions, focusing on the key problems to be solved and formulating an intervention strategy for the implementation of a scientific exercise program for community-dwelling elderly people with frailty and prefrailty to provide a reference for the next steps of formulating a sustainable implementation plan. On the basis of the CFIR framework, this study proposes the following strategies: The community smart health hut platform can be used to manage an integrated intelligent and personalized exercise prescription program in the home community. The community can actively organize community sports and health education lectures to support families, communities and involved peers to improve elderly people’s self-efficacy in sports. A community exercise intervention team should be established, responsibilities should be clarified, professional advantages should be fully utilized, and training in professional knowledge of sports and the implementation of sports programs should be provided. Frailty is a serious public health problem, and there are challenges to the implementation of exercise programs in this population. However, the implementation of science-based sports programs can improve the frailty and quality of life of older people in the community by creating a supportive internal and external exercise environment in multiple sectors, including government and primary healthcare services.

## Individual factors: promotion of health education for elderly people in the community with prefrailty to improve self-efficacy

Community sports and health education lectures should be actively promoted. Before activities are conducted, the community should issue activity notices on multiple platforms to expand the scope and try to ensure that elderly people in surrounding and even remote communities receive the notices. The activities should be rich in content, illustrated and easy to understand so that they are suitable for older people of different ages and knowledge levels. In places where activities are often performed, posters on the positive effects of exercise should be posted to provide information about the importance and benefits of exercise and to teach elderly people how to test their own exercise endurance and choose an exercise method that can ensure their safety. This will allow them to perceive the benefits of exercise and a sufficient sense of security to improve its effects. Supporting families, communities, and peers, improving the exercise self-efficacy of elderly people, helping frail and prefrail elderly people develop good exercise habits, and providing peer education promotes not only the sharing of exercise knowledge and the mastery of motor skills among frail and prefrail elderly people but also imitation and potential competitiveness among peers while improving attitudes and motivations toward exercise compliance as well as the ability to self-manage exercise.

## Internal factors: constructing an intelligent exercise prescription management plan for the home community

Community health service organizations should actively conduct comprehensive assessments and precise management of the frailty of elderly people, use the internet and information technology to integrate prevention and treatment systems, form a closed loop of healthcare management in communities and hospitals, actively promote community smart health hut platforms, and adopt individualized and standardized comprehensive diagnosis, treatment and intervention services to achieve people-oriented, all-round, full-cycle health management. Through a family physician service program with older adults in the community, internet-based tools and questionnaires can be used to collect comprehensive information on age, sex, physical mobility, healthy lifestyle, chronic disease history, and medication history while analyzing individual circumstances to support decision-making about personalized exercise prescriptions. All exercise prescription recommendations should be based on clinical exercise guidelines and the guidelines of the American Academy of Sports Medicine and Asia–Pacific region. Intelligent decision-making technology can map entities in the knowledge graph to corresponding spaces, obtain a vector representation of the entities and relationships in the graph to calculate the similarity between users, and construct a medical knowledge graph for exercise prescription. Frailty-related exercises can be recommended, and personalized exercise prescriptions can be generated on the basis of the comprehensive information entered in the early stage about individual exercise habits. In this study, the investigators issued exercise prescriptions to the participants, explained the exercise content and precautions, and followed up with the participants on a weekly basis.

## External factors: building a supportive exercise environment for frail community-dwelling elderly people in the context of healthy aging

Maximizing the functioning of older people in a healthy and supportive environment is the key to achieving healthy aging. In 2019, the National Health Commission issued the “Healthy China action (2020)”, which proposed the need to improve the health service system for elderly people, improve home and community care policies, create a liveable environment for elderly people, and achieve healthy aging. In 2021, The State Council Issued The National Fitness Plan (2021), which proposed improving the suitability of fitness facilities, sports fitness and leisure projects, and events for elderly people; creating barrier-free environments for public fitness facilities; conducting rehabilitation and fitness activities; preventing the occurrence of chronic diseases in elderly people; and promoting healthy aging. Frailty is a serious public health problem that requires society-wide efforts to slow its progression through the use of exercise interventions at the primary healthcare level. In the context of healthy aging, national basic public health services can be used to strengthen the construction of basic sports facilities and venues in the community, create a supportive sports environment, formulate public sports strategies, and actively build healthy communities.

## Implementing interventions

### Research subjects

This study was carried out in the two communities of Dongfeng and Hubin in Bengbu City, which represent the urban‒rural distribution of the elderly population in Bengbu City. These two community health service centers have previously cooperated with universities and have high levels of medical services. Community doctors, nurses, volunteers, and rehabilitation physiotherapists were the key implementation groups. From 2023 to 2024, health examinations were carried out in two major communities, and the resident elderly individuals with debilitating symptoms were selected as the main assessment objects. The inclusion criteria were as follows: (1) aged ≥60 years; (2) older adults who met the criteria for frailty on the basis of screenings and assessments; (3) able to use smartphones normally; and (4) provided informed consent and voluntarily participated in this study. The exclusion criteria were as follows: (1) a diagnosis of severe cognitive impairment or psychiatric disorders and (2) unwillingness or inability to participate consistently in follow-up interventions. Elderly people in the Dongfeng and Hubin communities were selected as the research subjects, with elderly people in the Hubin community comprising the control group, and elderly people in the Dongfeng community comprising the intervention group. The distance between communities reduced communication between neighbors and minimized ethical issues.

### Sample size calculation and grouping

According to the “Medical Research Methods” edited by Chen Shiyao et al. (Chen and Liu, 2015), the formula for calculating the sample size is N1 = N2 = 2×{(μα+μβ)σ/δ}^2^. According to the TFI score (Zhu, 2021) in the previous literature, the score δ was 0.12, the standard deviation σ was 0.09, α = 0.05, and β = 0.01. The two-sided test calculated the sample size of each group as follows: N = 2×{(1.96 + 2.58)×0.09/0.12}^2^ ≈ 30, considering a loss rate of 10%–20%. The sample size of each group was at least 36, and the final sample size was 80 cases, with 40 cases in the control group and 40 cases in the intervention group. Propensity score matching of 1:1 was used to match individuals in the control group with those in the intervention group in terms of the covariates. The number of people in the two groups after matching was determined. Each of the two groups included 22 community workers (4 doctors, 10 nurses, 2 rehabilitation physiotherapists and 6 volunteers).

## Research tools

### General information questionnaire

By querying various books and electronic information databases related to exercise interventions for frailty in elderly people at home and abroad as well as research in various databases, including books, journals, master’s and doctoral theses, newspapers and periodicals, the relevant variables were comprehensively identified, and a general data questionnaire was compiled. The general information questionnaire included sociodemographic and clinically relevant data. Sociodemographic variables included age, sex, marital status, education, hukou type, self-rated health status, smoking and drinking status, and economic status. The clinical data were related mainly to the comorbidities of the elderly individuals.

### Frailty assessment scale

The Dutch scholar Gobbens et al. (Gobbens et al., 2012) discussed the effectiveness of the Tilburg Frailty Indicator (TFI) and its application in intervention research. The Chinese scholar Xi Xing (Xi et al., 2013) developed the English version of the Tilburg Frailty Assessment Scale. The Cronbach’s alpha (coefficient of 0.686) of the Chinese version of the scale has acceptable reliability and validity. As a self-rating scale, the Tilburg Frailty Assessment Scale assesses the physical and mental condition of elderly people in three dimensions, namely, physical frailty, psychological frailty and social frailty, with 15 items. The total score ranges from 0 to 15 points: 0 points indicates normal; a score of 1‒4 indicates the predebilitating period; a score of ≥5 indicates frailty; and higher scores indicate greater frailty (Balasch-Bernat et al., 2023).

### Outcome indicators

The outcomes of this study mainly included implementation outcomes, process outcomes, intervention outcomes, and resource utilization. Implementation outcomes mainly include the feasibility, acceptability, and fidelity of community workers’ implementation of exercise intervention strategies (Proctor et al., 2011). The intervention outcomes mainly included physical fitness, psychological state, social support, and self-efficacy. Process outcomes mainly refer to indicators that ensure that the desired effect can be achieved during the implementation process, such as motor skill knowledge. In addition, the resource utilization of intervention strategies needs to be evaluated.

Feasibility: Refers to the extent to which an intervention strategy has been successfully implemented in a given setting (Proctor et al., 2011). In this study, community workers were surveyed and followed up to assess whether the implementation strategy was practical or suitable for daily use.

Acceptability: Refers to the degree of satisfaction or credibility of stakeholders with the content, process, services, *etc.*, of the implementation of the strategy (Proctor et al., 2011). The visual analog scale (VAS) (Yan, 2014) scoring method was used to evaluate the acceptance of exercise implementation strategies by social workers and frail older adults. The operator uses a 10 cm horizontal line on the ungraduated paper as a scoring criterion, and the score ranges from 0 to 10 from the left end to the right end. The closer the value is to 10, the greater the acceptability.

Fidelity: Refers to the degree to which the intervention strategy is implemented as designed by the original protocol or plan (Proctor et al., 2011). This study assessed the adherence to and completeness of social workers’ exercise strategy implementation through video observations or self-reports.

Physical fitness: Mainly includes muscle strength, muscular endurance, balance, body composition, *etc.* In this study, the testing instruments provided by the Hefei Intelligent Research Institute of the Chinese Academy of Sciences were used to measure the gait speed, one-leg standing balance test, grip strength, BMI, body fat percentage, *etc.*, of elderly individuals.

Psychological indicators: The Anxiety Self-rating Scale (SAS) was used to evaluate the anxiety level of the elderly individuals in the two groups, with a cutoff value of 50. A higher score indicates more severe anxiety (Cheng et al., 2023). The self-rating depression scale (SDS) was used to assess the degree of depression in both groups of older adults, with a cutoff score of 10 and higher scores indicating greater severity (LIU M. et al., 2023).

Social support: The Perceived Social Support Scale (PSSS) includes three dimensions, family support, friend support, and other support, with a total score of 84 points. Higher scores represent more social support received (CHEN et al., 2024).

Self-Efficacy: The exercise self-efficacy scale was developed by Resnick and Jenkins (2000). The Chinese version of the scale was developed by Zhang et al. (2016) and has a reliability of 0.87 and validity of 0.96. According to the difficulties encountered in the exercise process, the scale has a total of 9 items rated from 0 to 10, where “0” indicates “no confidence at all” and “1” indicates “very confident”. The ratings for all the items were added and divided by the number of items to score the participants’ exercise self-efficacy. A higher score indicates greater exercise self-efficacy for the participant.

Process indicators: A standardized exercise intervention was employed according to clinical implementation guidelines. The equipment for the exercises, such as elastic bands, barbells, grip machines, etc., were purchased and knowledge and skills assessments were conducted among the medical staff and community volunteers (out of 100 points). The equipment purchased and the level of knowledge and skills of the medical staff and community volunteers are all indicators of the process of implementing this strategy.

Resource utilization: Refers to resource usage and cost consumption. Microcost analysis was used to assess the costs and resource use of the interventions.

## Implementation of the interventions

### The control group received routine care

The main content included the following. (1) The Lakeside Community Health Service Center provided exercise-related knowledge lectures for frail and prefrail elderly individuals. Multimodal physical exercises, including warm-up activities, aerobic activities, flexibility training, and balance training, as well as the prescription of rehabilitative exercises, was carried out twice a week for 12 weeks for 60 min each time under the guidance of professional nursing staff. (2) The “Exercise Guidance Manual for Prefail Elderly Individuals in the Community”, which was compiled by the researchers, was made available. (3) Regular one-on-one telephone follow-up visits were conducted by graduate students. (4) Health check-ups were provided by community nurses.

### The intervention group received a scientific nursing intervention

In accordance with the Chinese version of the Tilburg Frailty Assessment Scale, the frailty of elderly people in the community was investigated, and an integrated development path for the construction of smart health huts and health promotion was explored and analyzed. The new needs of elderly people in our community hospital were considered to increase the exercise methods and medical-body collaboration, and a framework of online and offline exercise programs was constructed for elderly people in the early stages of frailty and prefrailty. With respect to offline programs, the instrument was used to detect frailty and prefrailty in elderly people according to their assessment data, living habits and exercise status to automatically generate a personalized exercise prescription. The exercise prescription included a specific amount, type, intensity, frequency, and time of exercise; the recommended type of pictures; and screening for risks and incorrect exercise-related behaviors corresponding to exercise precautions to provide regular benefits. Furthermore, behavioral guidance, risk prevention and control and other health education were provided, health promotion reports were generated, and oversight and feedback were provided.

With respect to the online program, hardware and software connected by computers, cloud services and mobile clients were used to implement the interventions, monitor the participants and provide feedback. We established a WeChat public account and regularly maintained, published, and updated relevant content about physical exercise, including the key points, the frequency and strength of the exercises, the weight-bearing time and resistance and solutions for problems encountered during the exercises. Audio, video, and animations were included. Online answers were provided to questions encountered during implementation.

### Quality control

This was a population study. Therefore, subjective and uncontrollable factors affected the results. It was necessary to strictly control the quality of each link and to control bias. (1) Selection bias control: The enrollment of participants strictly adhered to the inclusion and exclusion criteria to ensure that the study population was representative. (2) Information bias control: The researchers involved in the study were strictly trained, the investigation criteria and procedures were unified, and the criteria for frailty or prefrailty were confirmed by the scale. (3) Confounding bias control: The study design was reasonable, and the propensity score matching method was used to allocate participants to the control group and the intervention group so that the nontreatment factors and confounding factors of the study subjects were balanced. This approach helped to minimize the potential impact of confounding factors on the analysis of intervention effectiveness and enhances the credibility of the research results. (4) Loss to follow-up bias control: A good cooperative relationship was established with the research subjects, patients’ questions were addressed in a timely manner, trust was built, and loss of research subjects was prevented.

### Statistical methods

SPSS 26.0 software was used to analyze the survey and test data statistically. Continuous data are expressed as the mean ± standard deviation (x ± s), and the chi-square test and the rank sum test were used for comparisons of baseline data between groups. T tests were used for normally distributed continuous data, and the nonparametric test was used for nonnormally distributed data. P < 0.05 was considered to indicate statistical significance.

## Results

### General information on the research subjects

A total of 80 elderly people were included in this study at baseline. Four of them withdrew from the intervention due to illness or other reasons, and 8 of them did not adhere to the 3-month exercise plan. In this study, 32 pairs were successfully matched *via* 1:1 proximity matching, including 34 males and 30 females, with an average age of 69.08 ± 5.91 years. All baseline data between the two groups were balanced. There were no statistically significant differences between the intervention group and the control group in terms of baseline characteristics or implementation indicators, suggesting that the two groups were comparable, as shown in Table 4.

**TABLE 4:** Comparison of the two groups of data after matching.

Items	Intervention group (n = 32)	Control group (n = 32)	*t/*χ^2^	P
Age	68.03 ± 5.42	66.06 ± 5.25	1.475	0.145
Gender	​	​	0.000	1.000
Male	17 (53.13)	17 (53.13)	​	​
Female	15 (46.87)	15 (46.87)	​	​
Residence	​	​	0.611	0.434
Rural	19 (59.38)	22 (68.75)	​	​
City	13 (40.62)	10 (31.25)	​	​
Marital status	​	​	0.110	0.740
With partner	27 (84.38)	26 (81.25)	​	​
Unaccompanied	5 (15.62)	6 (18.75)	​	​
Education/year	​	​	0.071	0.790
≤9	21 (65.63)	22 (68.75)	​	​
>9	11 (34.37)	10 (31.25)	​	​
Economic	​	​	0.254	0.614
Low-middle	17 (53.13)	19 (59.37)	​	​
High	15 (46.87)	13 (40.63)	​	​
Smoking	​	​	0.254	0.614
Yes	13 (40.62)	15 (46.87)	​	​
No	19 (59.38)	17 (53.13)	​	​
Drinking	​	​	1.164	0.281
Yes	24 (75.00)	20 (62.50)	​	​
No	8 (25.00)	12 (37.50)	​	​
Comorbidities	​	​	2.259	0.133
Yes	18 (56.25)	12 (37.50)	​	​
No	14 (43.75)	20 (62.50)	​	​
Self-rated health	​	​	0.366	0.545
Fair or bad	26 (81.25)	24 (75.00)	​	​
Good	6 (18.75)	8 (25.00)	​	​
Gait speed	3.80 ± 1.46	4.51 ± 2.63	1.575	0.187
Balance	6.59 ± 1.12	6.43 ± 1.31	0.335	0.635
Grip strength	29.69 ± 9.95	28.99 ± 8.09	0.213	0.832
BMI	22.53 ± 5.07	23.35 ± 4.12	0.154	0.878
Body fat %	25.73 ± 7.69	24.37 ± 7.46	1.607	0.113
Anxiety	45.63 ± 11.32	42.25 ± 11.02	1.208	0.231
Depression	47.59 ± 8.17	46.31 ± 5.10	7.150	0.902
Social support	41.25 ± 12.75	45.50 ± 15.48	3.879	0.282
Exercise self-efficacy	6.76 ± 1.69	4.39 ± 1.78	5.009	0.129

### Comparison of the effects of the intervention program

#### Implementation results

The control group was given a routine nursing intervention, and the intervention group was implemented on the basis of routine nursing to carry out a scientific exercise strategy intervention. There were statistically significant differences in feasibility, acceptability and fidelity between the two groups after the intervention (P < 0.05). At the implementation level, all community workers in the intervention group participated in all the implementation strategies as planned, with a 100% attendance rate. At the intervention level, the compliance of only 2 older adults gradually decreased with the duration of the intervention. See Table 5.

**TABLE 5:** Comparison of the outcomes between the two groups after 3 months of intervention.

Items	Practice subjects	Total	Intervention group	Control group	χ^2^/t	P
Feasibility	Community workers	44	95.5% (21/22)	54.5% (12/22)	9.818	0.002
Acceptability	Community workers	44	9.3 ± 0.6	7.6 ± 0.4	0.019	0.003
	Intervention targets	64	9.7 ± 0.8	6.9 ± 0.6	0.024	0.006
Fidelity	Community workers	44	100% (22/22)	59.1% (13/22)	11.314	0.001
	Intervention targets	64	93.8% (30/32)	65.6% (21/32)	7.819	0.005

#### Intervention results

##### Physical fitness

The gait speed, single-leg standing balance test score, grip strength and body fat percentage were significantly improved in the two groups before and after the intervention (P < 0.05), as shown in Table 6. The improvements in gait speed, single-leg standing balance test results, grip strength and body fat percentage in the frail and prefrail elderly individuals in the intervention group were greater than those in the control group (P < 0.05), as shown in Table 7.

**TABLE 6:** Comparison of physiological indexes between the two groups before and after intervention.

Items	Intervention group				Control group			
	Pre-intervention	Post-intervention	χ^2^/t	P	Pre-intervention	Post-intervention	χ^2^/t	P
Gait speed	3.80 ± 1.46	5.91 ± 1.91	2.962	<0.001	4.51 ± 2.63	6.91 ± 2.87	3.040	0.012
Balance	6.59 ± 1.12	10.59 ± 0.97	1.569	<0.001	6.43 ± 1.31	7.59 ± 1.59	1.616	0.007
Grip strength	29.69 ± 9.95	37.81 ± 11.42	2.237	0.001	28.99 ± 8.09	35.65 ± 10.57	2.850	0.005
BMI	22.53 ± 5.07	23.46 ± 4.32	0.309	0.053	23.35 ± 4.12	23.86 ± 4.43	0.024	0.453
Body fat %	25.73 ± 7.69	23.42 ± 6.81	5.396	<0.001	24.37 ± 7.46	23.70 ± 6.02	2.027	0.043

**TABLE 7:** Comparison of physiological indexes between the two groups after 3 months of intervention.

Items	Intervention group	Control group	P
Gait speed	6.91 ± 2.87	5.91 ± 1.91	0.016
Balance	10.59 ± 0.97	7.59 ± 1.59	<0.001
Grip strength	37.81 ± 11.42	35.65 ± 10.57	<0.001
BMI	23.46 ± 4.32	23.86 ± 4.43	0.676
Body fat %	23.42 ± 6.81	23.70 ± 6.02	<0.001

##### Psychological indicators and social support

The depression and social support levels of the elderly individuals in the intervention group were significantly improved before and after the intervention (P < 0.05), and the depression level in the control group was significantly improved before and after the intervention (P < 0.05); however, there was no significant difference in anxiety or social support levels (P > 0.05), as shown in Table 8. The improvement in the depression and social support levels among the frail and prefrail elderly individuals in the intervention group were greater than those of the control group (P < 0.05), as shown in Table 9.

**TABLE 8:** Comparison of psychological indicators and social support indicators between the two groups after 3 months of intervention.

Items	Intervention group				Control group			
	Pre-intervention	Post-intervention	χ^2^/t	P	Pre-intervention	Post-intervention	χ^2^/t	P
Anxiety	45.63 ± 11.32	39.21 ± 10.39	3.751	0.765	42.25 ± 11.02	41.21 ± 11.00	3.260	0.556
Depression	47.59 ± 8.17	34.22 ± 7.37	4.777	<0.001	46.31 ± 5.10	44.22 ± 5.06	4.196	<0.001
Social support	41.25 ± 12.75	53.09 ± 14.15	5.405	<0.001	45.50 ± 15.48	44.81 ± 14.25	3.166	0.944

**TABLE 9:** Comparison of psychological and social support indicators between the two groups after 3 months of intervention.

Items	Intervention group	Control group	P
Anxiety	39.21 ± 10.39	41.21 ± 11.00	0.786
Depression	34.22 ± 7.37	44.22 ± 5.06	<0.001
Social support	53.09 ± 14.15	44.81 ± 14.25	<0.001

##### Exercise self-efficacy

The exercise self-efficacy scores were significantly improved before and after the intervention in the two groups (P < 0.05), as shown in Table 10. The exercise self-efficacy scores of the frail and prefrail elderly individuals in the intervention group were better than those of the control group (P < 0.05), as shown in Table 11.

**TABLE 10:** Comparison of self-efficacy scores between the two groups before and after the intervention.

Items	Intervention group				Control group			
	Pre-intervention	Post-intervention	χ^2^/t	P	Pre-intervention	Post-intervention	χ^2^/t	P
Self-efficacy	6.76 ± 1.69	8.31 ± 0.70	1.762	<0.001	4.39 ± 1.78	6.76 ± 1.29	1.538	0.009

**TABLE 11:** Comparison of self-efficacy scores between the two groups before and after intervention.

Items	Intervention group	Control group	P
Self-efficacy	8.31 ± 0.70	6.76 ± 1.29	<0.001

##### Process indicators

All practice subjects were given standardized exercise interventions according to clinical implementation guidelines. During this period, a total of 80 elastic bands, 40 barbells, and 40 grip devices were purchased, and all the equipment required for the exercises were able to meet the exercise needs of the practice subjects. Moreover, the knowledge and skill assessment scores of the medical staff and community volunteers in the two groups improved during the intervention process, and the knowledge and skill assessment scores of the intervention group were significantly higher than those of the intervention control group (P < 0.05), as shown in Tables 12, 13.

**TABLE 12:** Comparison of knowledge and skill assessment scores between the two groups before and after intervention.

Items	Intervention group				Control group			
	Pre-intervention	Post-intervention	χ^2^/t	P	Pre-intervention	Post-intervention	χ^2^/t	P
Knowledge and skill	61.4 ± 7.23	83.1 ± 7.79	1.762	<0.001	58.9 ± 6.76	75.6 ± 7.29	1.538	0.009

**TABLE 13:** Comparison of knowledge and skill assessment scores between the two groups after 3 months of intervention.

Items	Intervention group	Control group	P
Knowledge and skill	83.1 ± 7.79	75.6 ± 7.29	<0.001

##### Resource utilization

In terms of environmental resources, we actively exploited the cooperation between colleges and universities to carry out exercise intervention strategies in two community hospitals. In terms of material and financial resources, the purchased sports equipment was simple to use and inexpensive, but highly functional, which ensured the effectiveness and safety of the implemented sports program and increased the convenience for the community workers. In terms of manpower, the community smart health hut platform is free for assessing frailty and reduces the pressure of questionnaire collection. Moreover, the formation of standardized exercise intervention groups reduces the consumption of human resources and improves the compliance with and integrity of the implementation strategies.

## Discussion

### The implementation of an exercise strategy based on the CFIR framework is scientifically feasible and scalable

The CFIR framework is among the most widely used theoretical frameworks in the field of implementation research. It is used to identify obstacles and facilitators in multidimensional and context-specific situations in the process of implementing interventions and then to develop targeted implementation strategies (Kuo et al., 2022). This study used the CFIR framework to construct an exercise implementation strategy for elderly people in communities with frailty or prefrailty to maximize its application in practice. After the practice, the awareness and skill level of the community workers of exercise interventions for elderly patients with frailty improved, the operation related to the exercise interventions was standardized, and the exercise safety of frail elderly individuals was effectively guaranteed. The processes, systems and tools identified by the practice strategy were gradually integrated into the community work system, and a standardized intervention model and system were formed so that it can be sustained. In addition, this study analyzed the promoting and hindering factors in the implementation process, optimized local community resources and technologies, improved resource utilization, and actively created and used an external support environment, which effectively improved compliance with the implementation of community movement strategies, improved the physical fitness, depression, social support and self-efficacy of frail and prefrail community-dwelling elderly people, and delayed the progression of frailty. Through empirical research, the scientific feasibility and generalizability of the CFIR framework-based exercise interventions for elderly people with community frailty and prefrailty were confirmed.

### The implementation of scientific exercise interventions can effectively improve the physical fitness of frail or prefrail elderly individuals

On the basis of the CFIR framework, this study identified a variety of influencing factors in the implementation process through qualitative interviews, including external factors (lack of individualized exercise methods, etc.), internal factors (peer effects, sports atmosphere, lack of information technology facilities for teaching, limited access to exercise knowledge, etc.), and individual factors (lack of knowledge and skills related to regular exercise). On this basis, implementation strategies for building integrated and intelligent home- and community-based exercise prescription management plans and promoting community sports and health education were proposed. The results revealed that the gait speed, one-leg standing balance test, grip strength and body fat percentage results of the frail and prefrail elderly individuals in the intervention group were better than those in the control group. Physical fitness is a series of abilities or characteristics, including muscle strength, speed, balance, body composition, etc., that are manifestations of the functions of the various organ systems of the human body through physical activity (Zhang T. et al., 2021). With age, the body’s metabolism slows, muscle strength and endurance decrease, muscle fibers become thinner and decrease in number, and the central nervous system becomes dysfunctional, limiting exercise speed, balance, and strength (Kelley and Kelley, 2006). This study made full use of the community smart health hut platform to collect comprehensive information, such as age, sex, physical activity ability, exercise habits, and physiological function test results, such as cardiovascular, bone density, and arteriosclerosis results. This information was used to develop personalized exercise prescription recommendations through intelligent decision-making technology, including the exercise amount, exercise type, intensity, frequency, time, *etc.*, and pictures were included to provide behavioral guidance in the conduct of the recommended exercises to mitigate any potential risks associated with incorrect exercise-related behaviors and guide the participants on any potential precautions, automatically generating health promotion reports and effectively making up for the shortage of exercise methods skills and knowledge among the elderly. Compared with general exercise interventions, elderly individuals with different body characteristics can choose the most appropriate exercise method according to their own health condition, which is suitable for the physical condition of frail and prefrail individuals and can promote the physiological functioning of the body in a more targeted manner. Moreover, this study actively responded to national sports policies such as the Healthy China Action (2019–2030) and the National Fitness Plan (2021–2025) (Healthy China Action, 2020; The State Council Issued The National Fitness Plan, 2021), fully creating a supportive sports environment, carrying out community mobilization activities in the form of posters or banners, encouraging elderly individuals in the community to actively participate in sports and health actions and learn and master sports and health-related knowledge and skills, and promoting exercise among elderly individuals to improve their health status.

### The implementation of scientific exercise interventions can significantly improve depression and social support levels in frail or prefrail older adults

On the basis of the CFIR framework, this study used qualitative interviews to identify a variety of influencing factors in the implementation of exercise intervention programs, including external factors (e.g., limited publicity of activities via mass media) and internal factors (e.g., peer effects, the sports atmosphere, the family community and other aspects of support and encouragement). On the basis of this study, we identified a need to support families and communities as well as to consider the role of peers, to actively conduct community sports and health promotion activities and other implementation strategies, and to combine online and offline interventions. The results of the study revealed that the depressed state of the older adults in the intervention group was lower than that before the intervention and significantly lower than that in the control group, and the social support score was significantly better than that in the control group. Frail older people are prone to lower social support because of their limited ability to take care of themselves and their reluctance to go out for activities or communicate with others. Conventional exercise measures lack group activities and do not include the promotion of family and social support and regular exercise education lectures; thus, conventional exercise programs do not fundamentally promote the exercise compliance of elderly people, and some elderly people are unable or unwilling to actively participate in such interventions because of their own frailty. However, this study adopted a group activity strategy, which, compared with conventional individual interventions, increased the social communication network of frail older adults, enhance their interaction with others, helped them obtain professional guidance for participating in sports, reduced loneliness and increased social participation in the exercise program. This study focused on the supporting role of families and communities, encouraging family members to also participate and prompting community workers to provide full emotional support, which stimulated elderly individuals’ in participating in the exercise program. Furthermore, an online intervention could increase elderly individuals’ information exchange related to the activities and increase the sense of participation of the frail and prefrail elderly individuals through the sharing of their exercise experiences.

### The implementation of a scientific exercise intervention can improve self-efficacy in prefrail or frail elderly individuals

Under the guidance of the CFIR theoretical framework, this study explored the factors that influence exercise compliance in elderly people in the early stage of frailty with respect to external factors (e.g., relevant policy support), internal factors (e.g., peer effects and the exercise atmosphere), and individual factors (e.g., self-efficacy, exercise demand, attitudes and lack of motivation). This study implemented online and offline joint interventions so that elderly people could experience personalized, whole-process and systematic comprehensive management. This approach builds a bridge of trust between community health service personnel and elderly people and can help elderly people recognize and actively accept exercise programs and achieve a sense of self-identity. Supporting the encouragement and peer education of family members not only promotes the sharing of exercise knowledge and the mastery of motor skills among frail and prefrail elderly individuals but also helps these individuals develop good exercise habits. It can also promote imitation and potential competitiveness among peers, improve attitudes and motivation toward exercise compliance, and improve elderly people’s ability to self-manage exercise.

### Exercise intervention based on the CFIR framework can improve resource utilization and acceptability

The implementation strategy of this study successfully promotes the rational utilization of human, material and financial resources. In terms of manpower, a team of doctors, nurses, rehabilitation physiotherapists, and community volunteers was constructed to implement the exercise intervention, and the skills and knowledge acquired during the implementation process can help more elderly people participate in the exercise intervention, improve compliance with the exercise implementation strategy, and further improve the frailty of elderly individuals in the community. The contribution of the community volunteers to this project should not be underestimated, which effectively compensates for the shortage of human resources in the community and, at the same time, provides a certain understanding of the characteristics of the elderly population in the community and deep intuitive experience after actively participating in the project. This study was recognized and supported by community leaders, so in terms of leadership, the community leaders and teams jointly developed the training and assessment plans and undertook their supervision and coordination, which effectively promoted the efficient implementation of the exercise intervention strategy. In terms of material resources, the elastic bands, barbells, grip devices, etc., purchased could effectively help the elderly individuals carry out multicomponent exercises, including resistance exercises, and these sports devices are small in size, easy to operate, and improve the convenience and standardization of the operation of the exercise implementation program. The advantages of the university–university cooperation provided a suitable sports venue and a high-quality smart health hut platform for this study, which promoted the successful implementation of the exercise intervention strategy. In terms of financial resources, this study made full use of scientific research funds, and the sports equipment purchased was of high quality, affordable, and recyclable.

This implementation strategy was accepted by both the community workers and frail and prefrail older adults. First, the intervention in this study relied on a community health service platform to conduct the intervention activities in places where elderly people have lived for a long time, so they had a sense of belonging. At the same time, the community smart health hut had formal institutional certification and standardized management and relied on medical universities to provide the professional and technical support for the implementation of the scientific program. In the process of implementation, the elderly people received technical guidance on the exercises, mastered the knowledge necessary to perform the exercises themselves, received high-quality nursing services and improved their confidence in the community. Second, for the intervention, this study used the closed-loop management process, set up a sports intervention team, conducted training on professional sports knowledge and the content of the exercise program, clarified the responsibilities and professional advantages of the team, dynamically monitored the exercise status and risk assessment of the participants, and provided professional online answers to any questions raised by the community residents; all of these services were convenient, fast and free to the elderly residents. Further suggestions for improvements to the program in the next stage of development include adding a physical examination of the motor function of the elderly people in the community at different stages, the provision of one-to-one systematic exercise suggestions, and corrections to deviations in exercise behaviors.

### Strengths and weaknesses

This study is the first to explore the promoting and hindering factors of exercise program implementation in the frail or prefail elderly individuals in the community on the basis of the CFIR framework and to construct and implement a scientific exercise intervention strategy through empirical research in two communities. By relying on the integration of a community home and an intelligent health promotion platform to implement the scientific exercise intervention, the requirements of an aging population in the information age are met, which further improves and develops the knowledge base of the elderly. A more complete sports care program for medical alliance collaboration has been established, and the informatization of the urban smart fitness service platform service system has accelerated. However, there are also limitations to this study. First, the study population was limited to 64 frail or prefrail elderly people in the two major communities of Bengbu City. Furthermore, the intervention time was only 3 months, the sample size was small, and long-term indicators of the effect of the implementation of the program, such as quality of life, were not well reflected. Future studies should expand the scope of the study, extend the duration of the intervention, and further verify the long-term effects of implementing a scientific exercise intervention. Second, this study did not find that exercise interventions based on the CFIR framework can improve BMI and anxiety levels in frail older adults in the community. The reasons for these results may be as follows: The first is the specificity of the study population. There is a U-shaped association between BMI and frailty; for frail elderly individuals, maintaining a normal or high BMI may be a protective factor against adverse outcomes and can delay the occurrence and development of frailty. Weight loss or obesity are associated with a greater risk of frailty in older adults (Rietman et al., 2018). BMI is affected by multiple factors, such as body shape, bones, and muscle. It does not accurately reflect body fat distribution or recent weight changes. Second, the follow-up time was short. Anxiety is a painful experience that is not commensurate with the physical situation; it is an emotional response to a serious deterioration in the value characteristics of real or future things (Qiu, 2018). With the progression of the disease, people’s ability to perform daily activities gradually decreases, their lifestyle and social roles change, their adaptability weakens, and their own feelings cannot be fully expressed, which damages their personal abilities and self-confidence.This study only verified the effect of the exercise program in the context of community-dwelling older adults, and did not address the barriers and facilitators to the implementation and promotion of the program across different settings.Additionally, anxiety persists and gradually worsens over time. Therefore, future studies should fully consider the continuity of exercise when carrying out exercise interventions for frail elderly individuals with anxiety.

## Conclusion

Guided by the CFIR framework, this study deeply analyzed the promoting and hindering factors in four dimensions when implementing a scientific exercise intervention, including the intervention program, the internal and external environment, and individual factors, through qualitative interviews. Using this information, a scientific exercise intervention strategy for elderly people in the community with frailty and prefrailty was constructed, which effectively improved the physical fitness, psychological function, social support and self-efficacy of elderly people with frailty or prefrailty in the community. This finding also confirms that the exercise intervention strategy is feasible and worthy of sustained promotion. Future studies should expand the sample size of the population, extend the intervention time, and further verify the long-term effects of implementing scientific exercise interventions. The CFIR theoretical framework is highly important for the study of the factors that influence implementation in China, including the role of weakness and intrinsic capacity. In future research, the CFIR should be used as a theoretical framework to implement scientific exercise interventions for elderly people in the community with reduced intrinsic capacity to promote the transformation of evidence into clinical practice.

## Data Availability

The original contributions presented in the study are included in the article/supplementary material, further inquiries can be directed to the corresponding authors.
